# Venous Thromboembolism in Surgically Treated Esophageal Cancer Patients: A Provincial Population-Based Study

**DOI:** 10.1055/s-0042-1750378

**Published:** 2022-07-11

**Authors:** Gileh-Gol Akhtar-Danesh, Noori Akhtar-Danesh, Yaron Shargall

**Affiliations:** 1 Department of Surgery, McMaster University, Hamilton, Ontario, Canada; 2 School of Nursing, McMaster University, Hamilton, Ontario, Canada; 3 Department of Health Research Methods, Evidence, and Impact, McMaster University, Hamilton, Ontario, Canada; 4 Division of Thoracic Surgery, St. Joseph's Healthcare Hamilton, Hamilton, Ontario, Canada

**Keywords:** esophageal cancer, extended prophylaxis, venous thromboembolism, long-term survival

## Abstract

**Objective** Venous thromboembolism (VTE) is a major cause of morbidity and mortality in surgical patients. Surgery for esophageal cancer carries a high risk of VTE. This study identifies the risk factors and associated mortality of thrombotic complications among patients undergoing esophageal cancer surgery.

**Methods** All patients in the province of Ontario undergoing esophageal cancer surgery from 2007 to 2017 were identified. Logistic regression identified VTE risk factors at 90 days and 1 year postoperatively. A flexible parametric survival analysis compared mortality and survival up to 5 years after surgery for patients with and without a postoperative VTE.

**Results** Overall 9,876 patients with esophageal cancer were identified; 2,536 (25.7%) underwent surgery. VTE incidence at 90 days and 1 year postoperatively were 4.1 and 6.3%, respectively. Patient factors including age, sex, performance status, and comorbidities were not associated with VTE risk. VTE risk peaked at 1 month after surgery, with a subsequent decline, plateauing after 6 months. Adenocarcinoma was strongly associated with VTE risk compared with squamous cell carcinoma (SCC) (odds ratio [OR] 2.53, 95% confidence interval [CI] 1.38–4.63, *p* = 0.003). VTE risk decreased with adjuvant chemotherapy (OR = 0.58, 95% CI 0.36–0.94, *p* = 0.028). Postoperative VTE was associated with decreased survival at 1 and 5 years (hazard ratio = 1.57, 95% CI 1.23–2.00, *p* < 0.001).

**Conclusion** Esophageal cancer patients with postoperative VTE have worse long-term survival compared with those without thrombotic complications. Adenocarcinoma carries a higher VTE risk compared with SCC. Strategies to reduce VTE risk should be considered to reduce the negative impacts on survival conferred by thrombotic events.

## Graphical abstract

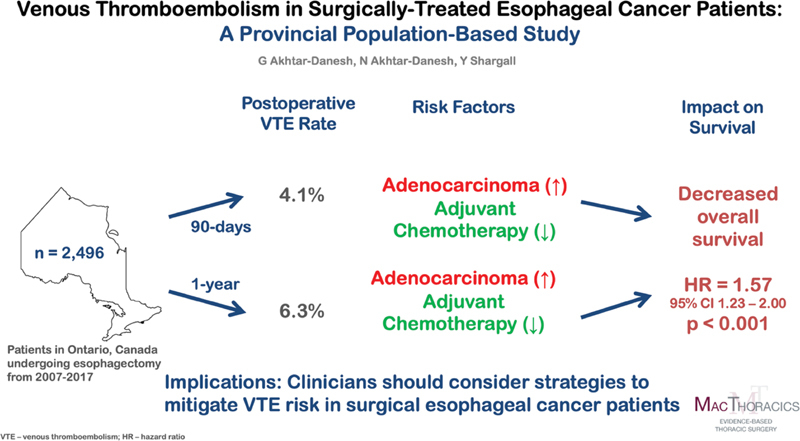

## Introduction

Venous thromboembolism (VTE), including deep vein thrombosis (DVT) and pulmonary embolism (PE), is a common complication in surgical patients resulting in significant morbidity, mortality, and resource utilization. A 2003 study identified VTE as the most preventable cause of morbidity and mortality in hospitalized patients in the United States, with an estimated annual cost of up to $10 billion. 1 2 The risk of VTE is heightened in cancer patients and even more so in those undergoing surgery for malignancy. 3 Accordingly, the American College of Chest Physicians (ACCP) and American Society of Clinical Oncology (ASCO) guidelines both recommend routine in-hospital thromboprophylaxis for patients undergoing cancer surgery. 4 5

In recent years, several surgical specialties including general surgical oncology and orthopaedics have developed recommendations for extended, postdischarge VTE prophylaxis, based on several randomized controlled trials. 6 7 However, evidence is still scarce regarding the role of extended prophylaxis in thoracic surgery. The current standard of care for thoracic surgery is in-hospital pharmacological and mechanical prophylaxis. 8 However, thoracic surgical patients represent a unique subset of surgical oncology with an increased VTE risk due to inherent technical and disease-specific factors. 9 10 Patients with esophageal cancer are at particularly high risk due to their underlying malignancy, decreased postoperative mobility, and the fact that the majority of esophageal cancer patients receive neoadjuvant chemoradiation, which has been reported as a risk factor for VTE. 9 11 12 Although based on a recent international Delphi survey most clinicians agree regarding the indications for extended prophylaxis in thoracic surgery, 13 the vast majority do not provide extended prophylaxis for patients post-esophageal cancer surgery. 14

The importance of extended VTE prophylaxis lies in the associated reduction in postdischarge thrombotic events. 15 Moreover, not only do thrombotic complications increase postoperative morbidity, but postoperative VTEs are also potentially associated with a drastic increase in mortality risk. 12 Therefore, identifying the subset of patients at highest VTE risk is of paramount importance to reducing the burden of thrombotic complications via administration of appropriate duration VTE prophylaxis.

This study examines the incidence and risk factors of postoperative VTE in patients undergoing surgery for esophageal cancer. Furthermore, the effect of thrombotic complications on short- and long-term survival will be examined. The results of this study can inform ongoing trails examining extended VTE prophylaxis in thoracic surgery patients, 16 and help elucidate the subset of esophageal cancer patients who may stand to benefit from extended prophylaxis.

## Materials and Methods

### Design

This study is a retrospective, population-based analysis of administrative data. All patients diagnosed with esophageal cancer in the Canadian province of Ontario from 2007 to 2017, with follow-up to the end of 2018, were identified. Ontario is Canada's largest province and comprises approximately 39% of its population. 17 Data were extracted from multiple population-based data sets maintained by the Institute for Clinical Evaluative Sciences (ICES). These data sets include all patients in Ontario with their age, sex, diagnosis, inpatient, and outpatient procedures. All patients aged 25 years or older at diagnosis were included in this analysis. Age at diagnosis was then classified as < 60, 60 to 69, 70 to 79, and 80 and over. The Ontario Cancer Registry was linked with other data sets to obtain oncologic information including neoadjuvant and adjuvant therapy, staging, pathology, type of surgical resection, and mortality. Variations in chemotherapy protocols (i.e., CROSS vs. FLOT) could not be tracked through this data. Staging information has been made available in Ontario since 2007. Cancer Care Ontario (CCO) uses a “best stage” grouping approach, where stage is assigned based on pathologic TNM (tumor, node, metastasis) when available, and clinical TNM otherwise. 18 The staging system in use at the time of diagnosis is entered by CCO. This study received ethics approval from the Hamilton Integrated Research Ethics Board (HiREB, number 7771-C). Written patient consent was waived as the study was completed with anonymized population-level administrative data

The development of a postoperative VTE was tracked through the Canadian Institute for Health Information Discharge Abstract Database. 19 Patients were followed for 12 months postoperatively to assess for VTE development, and up to 5 years after surgery to track survival and mortality. Of note, due to the nature of the data, it was not possible to track whether VTEs were diagnosed after development of symptoms, or by asymptomatic screening. Extended prophylaxis is currently not provided in Ontario to patients post-thoracic surgery. VTE events occurring postdischarge would have occurred off-treatment. Routine in-hospital chemical and mechanical prophylaxis is provided to esophageal cancer patients during hospital admission. This study did not track whether VTE events occurred during hospitalization versus postdischarge. ICES data are widely used across Ontario for clinical research and to guide health care delivery. 20

### Outcomes

The primary outcome of this study was the development of 90-day and 1-year postoperative VTE. In-hospital and postdischarge VTEs were both included. Any VTE occurring within 12 months after the index operation was captured. Patients with a history of VTE before surgery were excluded from the study. The 5-year survival rate after surgery was assessed as a secondary outcome.

### Predictors

Predictors of interest included patient, disease, and procedure-specific factors. Patient factors include demographic variables such as age, gender, comorbidities, Eastern Cooperative Oncology Group performance status, and frailty. Frailty was defined by the Johns' Hopkins Adjusted Clinical Groups (The Johns Hopkins ACG System), which is a validated instrument for use in administrative data sets. 21 Disease-specific factors include cancer histology, stage, chemoradiation (neoadjuvant, adjuvant, or curative), and disease site (esophagus and cardia). Finally, procedure-specific factors examined operative technique including minimally invasive esophagectomy (MIE) versus open surgery.

### Statistical Analysis

Descriptive statistics were used to characterize the patient population. The chi-square test was used to examine the association between outcomes of interest and categorical variables, while analysis of variance or Student's *t* -test was used for continuous variables. A multiple logistic regression was used to identify factors associated with VTE risk at 90 days and 1 year postoperatively. A flexible parametric survival (Royston–Parmar) model was used to estimate rate of VTE up to 6 months after surgery as well as 5-year survival rates based on VTE status adjusted for the aforementioned variables. 22 23 This model adopts a piecewise approach which is more flexible compared with other traditional methods in mimicking the actual trends in mortality (hazard rate) and survival pattern. 24 It provides smooth estimates of survival using restricted cubic splines on the log cumulative excess hazard scale. We fitted a model by incorporating all the abovementioned predictors and the interaction term between each two predictors. We also examined the time-varying effect of each predictor in the model. The likelihood ratio test was used to compare different models toward reaching a final model. All variables included in the final model were statistically significant ( *p* ≤ 0.05). Then, using the final model, the survival and mortality rates (per 100 person-months) were estimated while adjusting for the other variables. The local institutional ethics board approved the research proposal for this retrospective study.

## Results

Over the study period of 2007 to 2017, a total of 9,876 patients with esophageal cancer were identified in Ontario, of which 2,536 (25.7%) underwent surgical resection. Of these, 40 patients had a VTE diagnosed before surgery and were therefore excluded, resulting in a total study population of 2,496. Table 1 highlights the baseline characteristics of the patients included in this analysis. Staging information was available for 1,564 patients. Men comprised nearly 80% of the study population. The most common comorbidity was hypertension, which was seen in 1,354 patients. Of patients with known stage, stage II/III disease accounted for 76.7% of all esophagectomies, while 8.4% of esophagectomies were performed in patients with stage IV disease. The vast majority of esophagectomies were performed open, with only 16.7% undergoing MIE. The incidence of VTE at 90 days and 1 year postoperatively was 4.1% ( *n* = 101) and 6.3% ( *n* = 156), respectively.

**Table 1:** Baseline characteristics of patients with and without a postoperative VTE

	No VTE (%) b	VTE at 90 d (%)	VTE at 1 y (%) a	Total
Gender				
Male	1,868 (79.8)	85 (84.2)	126 (80.8)	1,994
Female	472 (20.2)	16 (15.8)	30 (19.2)	502
Age				
< 60	800 (34.2)	34 (33.7)	58 (37.2)	858
60–69	855 (36.5)	36 (35.6)	54 (34.6)	909
≥ 70	685 (29.3)	31 (30.7)	44 (28.2)	729
Year of diagnosis				
2007–2009	620 (26.5)	27 (26.7)	35 (22.4)	655
2010–2012	628 (26.8)	30 (29.7)	47 (30.1)	675
2013–2015	671 (28.7)	22 (21.8)	37 (23.7)	708
2016–2017	421 (18.0)	22 (21.8)	37 (23.7)	458
Comorbidities c				
Renal disease ± dialysis	26 (1.1)	0 (0)	0 (0)	26
COPD	473 (20.2)	21 (20.8)	30 (19.2)	503
Diabetes	565 (24.1)	30 (29.7)	50 (32.1)	615
Hypertension	1,264 (54.0)	59 (58.4)	90 (57.7)	1,354
Previous myocardial infarction	140 (6.0)	6 (5.9)	9 (5.8)	149
Frailty				
No	1,599 (68.3)	73 (72.3)	106 (67.9)	1,705
Yes	741 (31.7)	28 (27.7)	50 (32.1)	791
ECOG				
0	1,853 (79.2)	77 (76.2)	122 (78.2)	1,975
1	227 (9.7)	11 (10.9)	17 (10.9)	244
> 2	260 (11.1)	13 (12.8)	17 (10.9)	277
Stage				
I	224 (15.3)	7 (10.4)	9 (8.7)	233
II	485 (33.2)	19 (28.4)	34 (33.0)	519
III	629 (43.1)	34 (50.7)	51 (49.5)	680
IV	123 (8.4)	7 (10.4)	9 (8.7)	132
Histology				
Squamous cell carcinoma	361 (15.4)	9 (8.9)	12 (7.7)	373
Adenocarcinoma	1,576 (67.4)	79 (78.2)	126 (80.8)	1,702
Other	403 (17.2)	13 (12.9)	18 (11.5)	421
Disease site				
Cardia	889 (38.0)	43 (42.6)	64 (41.0)	953
Esophagus	1,451 (62.0)	58 (57.4)	92 (59.0)	1,543
Resection type				
MIE	388 (16.2)	17 (16.8)	29 (18.6)	417
Open esophagectomy	1,952 (83.4)	84 (83.2)	127 (81.4)	2,079
Radiation therapy				
No radiation	1,102 (47.1)	64 (63.4)	73 (46.8)	1,175
Curative	621 (26.5)	31 (30.7)	46 (29.5)	667
Neoadjuvant				
Adjuvant	617 (26.4) d	22 (21.8) d	37 (23.7) d	654
Chemotherapy				
No chemotherapy	930 (39.7)	55 (54.5) d	69 (44.2)	999
Curative	116 (5.0)		8 (5.1)	124
Neoadjuvant	791 (33.8)	38 (37.6)	56 (35.9)	847
Adjuvant	503 (21.5)	8 (7.9)	23 (14.7)	526
Total	2,340	101	156	2,496

Abbreviations: COPD, chronic obstructive pulmonary disease; ECOG, Eastern Cooperative Oncology Group; MIE, minimally invasive esophagectomy; VTE, venous thromboembolism.

a VTE at 1-year also includes VTE at 90-day.

b Reflects patients with no VTE recorded 1 year after surgery.

c Comorbidities were captured if patients had reports of underlying medical comorbidities for the 10 years preceding surgery.

d Due to a small number of VTE events (≤ 5), adjacent categories were combined to preserve patient anonymity.

The factors associated with VTE development at 90 days are highlighted in Table 2 . Interestingly, disease stage was not associated with VTE risk. However, adenocarcinoma conferred a 2.15 times higher risk of VTE compared with squamous cell carcinoma (SCC) (95% confidence interval [CI]: 1.07–4.34, *p* = 0.033). Furthermore, adjuvant chemotherapy seemed to decrease VTE risk by over 70% (odds ratio [OR] = 0.28, 95% CI: 0.13–0.59, *p* = 0.001).

**Table 2:** Factors associated with VTE risk at 90 days a

	Odds ratio	95% confidence interval	*p* -Value
Histology			
Squamous cell carcinoma	Reference	Reference	Reference
Adenocarcinoma	2.14	1.07–4.34	0.033
Other	1.39	0.59–3.31	0.454
Chemotherapy			
No chemotherapy	Reference	Reference	Reference
Curative	0.85	0.33–2.17	0.732
Neoadjuvant	0.88	0.56–1.35	0.550
Adjuvant	0.28	0.13–0.59	0.001

Abbreviation: VTE, venous thromboembolism.

a Results are from the multivariable model; in the interest of brevity only factors significantly associated with VTE risk are highlighted in the above table.

The factors associated with VTE risk at 1 year are presented in Table 3 . Once again, histology and adjuvant chemotherapy were the only factors associated with VTE risk. At 1 year after surgery, patients with esophageal adenocarcinoma had a 2.53 times higher risk of VTE compared with SCC ( *p* = 0.003). There was no association between resection type, disease stage, or comorbidities. Adjuvant chemotherapy again decreased VTE risk (OR = 0.58, 95% CI: 0.36–0.94, *p* = 0.028). There was no association between radiation and VTE risk.

**Table 3:** Factors associated with VTE risk at 1 year a

	Odds ratio	95% confidence interval	*p* -Value
Histology			
Squamous cell carcinoma	Reference	Reference	Reference
Adenocarcinoma	2.53	1.38–4.63	0.003
Other	1.40	0.67–2.97	0.370
Chemotherapy			
No chemotherapy	Reference	Reference	Reference
Curative	0.99	0.47–2.13	0.994
Neoadjuvant	0.93	0.65–1.35	0.709
Adjuvant	0.58	0.36–0.94	0.028

Abbreviation: VTE, venous thromboembolism.

a Results are from the multivariable model; in the interest of brevity only factors significantly associated with VTE risk are highlighted in the above table.

**Fig. 1:**
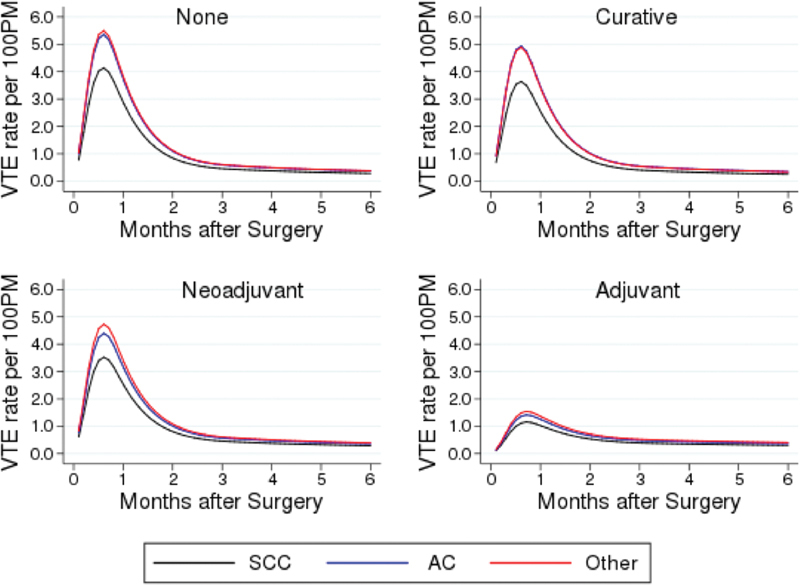
Adjusted risk of VTE development over time for all stages ( *p* < 0.002), and histology ( *p* for adenocarcinoma = 0.012). AC, adenocarcinoma; SCC, squamous cell carcinoma; VTE, venous thromboembolism.

The survival model for the development of VTE up to 6 months after surgery included histology of tumor and chemotherapy status. Also, the effect of chemotherapy was shown to be a time-varying effect. The model showed that the risk of VTE in esophageal cancer patients peaked at 1 month after surgery, with a subsequent gradual decline, eventually plateauing after 6 months ( Fig. 1 ). As highlighted above, adenocarcinoma carried the highest risk of VTE development. Perhaps most importantly, the development of a postoperative VTE was associated with the overall survival of esophageal cancer patients, regardless of other disease or patients-related factors.

**Fig. 2:**
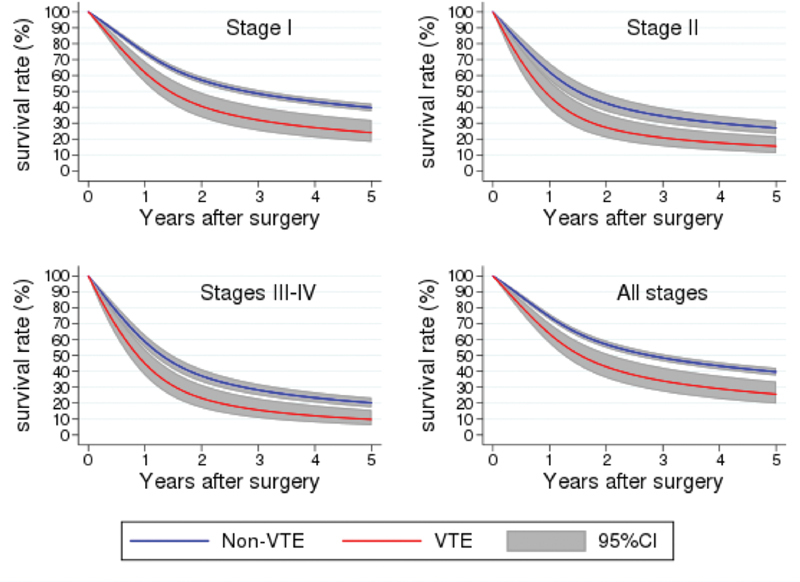
Adjusted survival for patients with and without a postoperative VTE ( *p* < 0.002). VTE, venous thromboembolism.

The final survival model which was used to estimate 5-year survival rates based on VTE status adjusted for patient, disease, and procedure-specific factors did not include VTE as a time-varying variable. The hazard ratio for 5-year survival for patients developing a VTE in the first year after surgery was 1.57 in comparison to patients without a thrombotic complication (95% CI: 1.23–2.00, *p* < 0.001). As the statistical model indicated, VTE reduced survival regardless of disease stage ( Fig. 2 ). Postoperative VTE development not only increased the mortality of esophageal cancer patients in the months after surgery, it reduced 5-year survival for all stages.

## Discussion

This study examined the patient, disease, and treatment-related factors associated with the development of postoperative VTEs in patients undergoing surgery for esophageal cancer. The effects of VTE development on overall survival were also assessed. We identified tumor histology to be associated with VTE development at both 90 days and 1 year, with adenocarcinoma conferring a higher risk. Adjuvant chemotherapy decreased VTE risk at both 90 days and 1 year. Patients who developed a postoperative VTE had a significantly higher mortality rate and reduced long-term survival compared with those without a postoperative VTE.

Patients undergoing cancer surgery have been well documented to carry a higher risk of VTE compared with their counterparts undergoing surgery for benign diseases. 25 Among cancer patients, several risk factors have been identified that further increase the risk of thrombotic complications. 26 Disease stage and histology are among the cancer-specific risk factors associated with VTE development. 27 Gastrointestinal adenocarcinomas are thought to carry the highest risk of thrombotic complications. 26 28 The findings of this study with respect to histology are consistent with the existing literature. Interestingly, however, this study did not find an association between disease stage and VTE risk. This is despite numerous studies having previously shown an association between increasing stage and risk of thrombotic complications. In a Danish population-based study, patients with advanced malignancies had a significantly higher risk of thrombotic events. 29 Similarly, in a prospective Italian registry advanced stage was a risk factor for DVT/PE. 25 While these studies examined a large group of cancer patients, the present analysis focused on esophageal cancer patients exclusively. Nevertheless, the lack of association between disease stage and VTE risk is an unexpected finding and is likely attributable to the small number of VTEs at each stage, suggesting insufficient power.

We also found that adjuvant chemotherapy was associated with a decreased risk of VTE development. This finding, which was consistent at 90-day and 1-year, is in contrast to the majority of existing literature. Chemotherapy has traditionally been viewed as an independent risk factor for VTE development. 28 The mechanisms by which chemotherapy increase VTE risk are well summarized in a review by Haddad and Greeno. 28 However, the literature surrounding chemoradiation-associated VTE risk in patients with esophageal cancer specifically remains conflicting, with some studies reporting no association between neoadjuvant therapy and VTE. Rollins et al conducted a prospective review of 200 patients undergoing curative treatment at a single center, and found a statistically significant increased risk of VTE in patients receiving neoadjuvant chemotherapy. 11 However, their single-center study only captured 27 VTE events, of which 12 occurred preoperatively, leaving a small number of postoperative events upon which to draw conclusions regarding postoperative VTEs. In contrast, Marshall-Webb et al conducted a narrative review of the literature and identified 14 studies examining the effects of neoadjuvant therapy on VTE risk. 30 Their study found an insufficient number of patients to reach statistically significant conclusions regarding the risks of neoadjuvant therapy on VTE risk. The largest study to date is a European series capturing nearly 3,000 patients with esophageal cancer and found no association between neoadjuvant therapy and VTE risk. 12 To our knowledge, our study is the first to report a negative association between chemotherapy and VTE development. Of note, while the aforementioned studies examined neoadjuvant therapy, our findings of a decreased association were only significant for chemotherapy in the adjuvant setting. This is another unexpected finding, as patients requiring adjuvant therapy are more likely to have advanced disease, in turn increasing VTE risk. We postulate that administration of chemotherapy following tumor resection might affect residual micrometastatic disease, diminishing the thrombogenic effect of the residual tumor cells. It is also possible that due to the retrospective nature of the study and the use of administrative data, we were unable to account for a strong confounder. Nevertheless, the association between chemoradiation and VTE risk in esophageal cancer patients requires further granular investigation.

Finally, our study found increased mortality and decreased long-term survival in patients developing thrombotic complications post-esophageal cancer surgery. VTE is one of the leading causes of death in cancer patients and is associated with a worse overall prognosis. 31 32 Even in patients with limited disease and curative intent resections, VTE remains a poor prognostic indicator, presumably indicative of more biologically aggressive tumors. 33 In our study, the hazard ratio for patients with a postoperative VTE was 1.57, and a decreased 5-year survival was seen for all stages. Both the ASCO Clinical Practice Guidelines and the ACCP 2012 guidelines recommend extended prophylaxis for patients undergoing abdominal or pelvic cancer surgery. 4 5 Although esophagectomy patients are considered high-risk in both guidelines, no specific recommendations for esophageal cancer patients are made. Numerous high-quality studies have demonstrated both the safety and efficacy of extended-duration prophylaxis in reducing the incidence of thrombotic events. 34 35 Furthermore, the present analysis clearly demonstrates a spike in VTE rates at 1 month after surgery, which is the time period targeted by extended-prophylaxis protocols. It should be noted that the present study did not capture cause of death, and therefore cannot make inferences regarding causality between VTE development and mortality. Nevertheless, there was a clear association between VTE and survival, which is in keeping with the existing literature. Given the high-risk nature of esophageal cancer surgery and the safety of extended-prophylaxis, the findings of this study strongly suggest considering extended VTE prophylaxis in patients post-esophagectomy. However, the type and duration of extended prophylaxis is yet to be determined based on adequately powered prospective trials.

This study has several strengths. First, we were able to capture all patients in the province of Ontario, Canada's largest province comprising approximately 39% of its population, 17 undergoing esophageal cancer surgery over a 10-year period. The linked ICES data sets contain high-quality detailed information on patient and cancer characteristics. Staging data and treatment information were available through CCO, allowing for an assessment of VTE risk based on several important disease-related factors. Survival was examined over an extended period, enabling an assessment of VTE-related mortality in the short-term, as well as its long-term effects on cancer survival. The use of a flexible parametric model for survival analysis allowed for a better estimation of actual survival compared with standard parametric models and Cox regression. The population-based nature of the study optimizes its external validity.

However, this study also has some limitations. Incomplete staging data limited the ability to analyze the entire cohort of esophageal cancer patients. The inherent limitations of administrative data meant that the complexity of treatment decisions including patient and/or health care provider preference could not be captured, and that the granularity of data are limited, as is the case in the vast majority of population-based database-driven analyses. For example, indications for adjuvant therapy could not be tracked in this study. Furthermore, while scoring systems such as the Caprini Risk Model and Khorana score have previously been studied and found to be associated with VTE risk, this level of data granularity is unavailable in the data sets employed, and therefore comparisons to other risk models cannot be drawn. All administrative data are subject to coding error, which may have impacted the findings relating to chemotherapy and radiation. Treatment decisions regarding anticoagulation and their complications after VTE diagnosis were not tracked; neither were the effects of anticoagulation treatment on survival, as medication changes can be unreliable in administrative databases.

In summary, esophageal cancer patients with adenocarcinoma histology have an increased risk of developing postoperative VTEs. The development of thrombotic complications is associated with the short-term mortality and long-term survival of esophageal cancer patients. Therefore, clinicians should consider strategies to mitigate this increased risk, including extended VTE prophylaxis, in high-risk patients undergoing surgery for esophageal cancer.

## Notes

**What is known about this topic?**
Surgical patients and in particular patients undergoing cancer surgery are at high risk of developing thrombotic complications including VTE. Extended VTE prophylaxis is recommended in patients undergoing major abdominal surgery for cancer, but no such recommendations exist for thoracic surgery patients.
**What does this paper add?**
Patients with esophageal adenocarcinoma have a higher VTE risk compared with squamous cell carcinoma. Importantly, the development of a postoperative VTE is associated with a reduction in long-term survival.
